# 2-(3-Fluoro­phen­yl)-5-iodo-7-methyl-3-methyl­sulfinyl-1-benzofuran

**DOI:** 10.1107/S1600536812018697

**Published:** 2012-05-05

**Authors:** Hong Dae Choi, Pil Ja Seo, Uk Lee

**Affiliations:** a Department of Chemistry, Dongeui University, San 24 Kaya-dong, Busanjin-gu, Busan 614-714, Republic of Korea; b Department of Chemistry, Pukyong National University, 599-1 Daeyeon 3-dong, Nam-gu, Busan 608-737, Republic of Korea

## Abstract

In the title compound, C_16_H_12_FIO_2_S, the 3-fluoro­phenyl ring makes a dihedral angle of 34.93 (7)° with the mean plane [r.m.s. deviation = 0.019 (1) Å] of the benzofuran fragment. In the crystal, mol­ecules are linked *via* pairs of I⋯O contacts [3.088 (2) Å] into inversion dimers. These dimers are connected by weak C—H⋯O hydrogen bonds.

## Related literature

For background information and the crystal structures of related compounds, see: Choi *et al.* (2008 ▶, 2010 ▶). For a review of halogen bonding, see: Politzer *et al.* (2007 ▶).
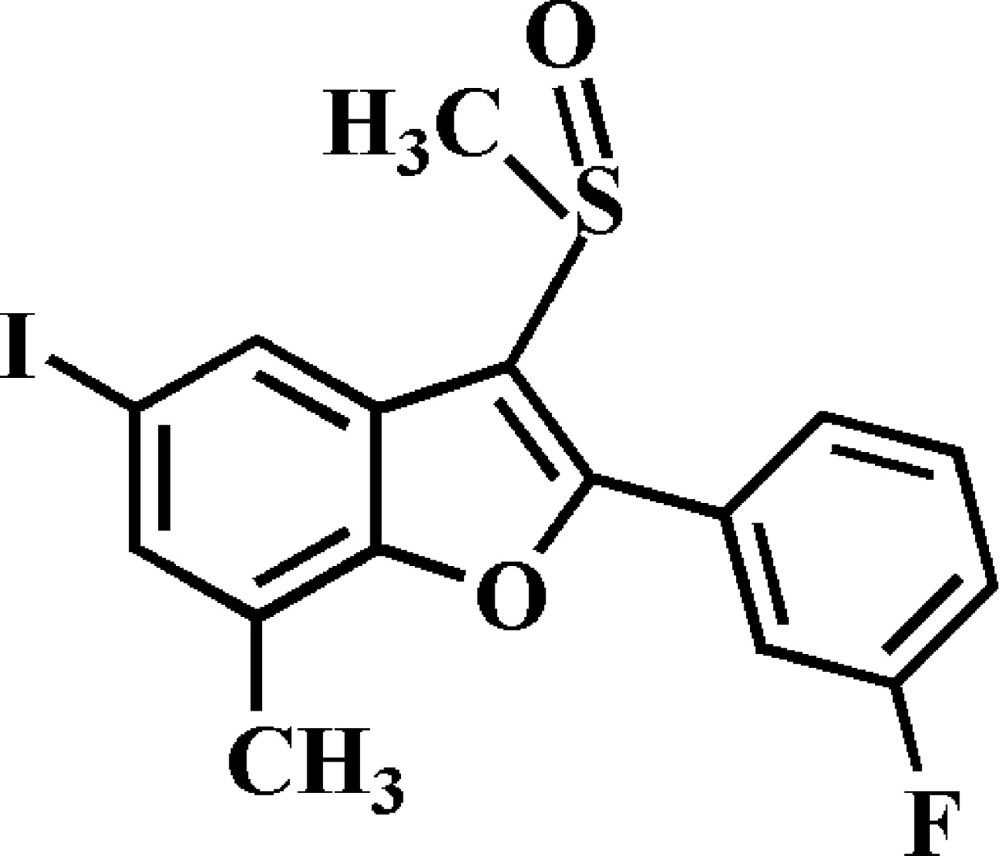


## Experimental

### 

#### Crystal data

C_16_H_12_FIO_2_S
*M* *_r_* = 414.22
Monoclinic, 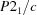

*a* = 7.8169 (1) Å
*b* = 23.9721 (3) Å
*c* = 8.0559 (1) Å
β = 99.748 (1)°
*V* = 1487.78 (3) Å^3^
*Z* = 4
Mo *K*α radiation
μ = 2.30 mm^−1^
*T* = 173 K
0.30 × 0.22 × 0.19 mm

#### Data collection

Bruker SMART APEXII CCD diffractometer
Absorption correction: multi-scan (*SADABS*; Bruker, 2009 ▶) *T*_min_ = 0.570, *T*_max_ = 0.746
14066 measured reflections
3424 independent reflections
3226 reflections with *I* > 2σ(*I*)
*R*_int_ = 0.025

#### Refinement

*R*[*F* ^2^ > 2σ(*F* ^2^)] = 0.021
*wR*(*F* ^2^) = 0.052
*S* = 1.10
3424 reflections
192 parameters
H-atom parameters constrained
Δρ_max_ = 0.45 e Å^−3^
Δρ_min_ = −0.55 e Å^−3^

### 

Data collection: *APEX2* (Bruker, 2009 ▶); cell refinement: *SAINT* (Bruker, 2009 ▶); data reduction: *SAINT*; program(s) used to solve structure: *SHELXS97* (Sheldrick, 2008 ▶); program(s) used to refine structure: *SHELXL97* (Sheldrick, 2008 ▶); molecular graphics: *ORTEP-3* (Farrugia, 1997 ▶) and *DIAMOND* (Brandenburg, 1998 ▶); software used to prepare material for publication: *SHELXL97*.

## Supplementary Material

Crystal structure: contains datablock(s) global, I. DOI: 10.1107/S1600536812018697/bt5901sup1.cif

Structure factors: contains datablock(s) I. DOI: 10.1107/S1600536812018697/bt5901Isup2.hkl

Supplementary material file. DOI: 10.1107/S1600536812018697/bt5901Isup3.cml

Additional supplementary materials:  crystallographic information; 3D view; checkCIF report

## Figures and Tables

**Table 1:** Hydrogen-bond geometry (Å, °)

*D*—H⋯*A*	*D*—H	H⋯*A*	*D*⋯*A*	*D*—H⋯*A*
C15—H15⋯O2^i^	0.95	2.44	3.183 (3)	135

Symmetry code: (i) 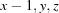.

**Fig. 1.**
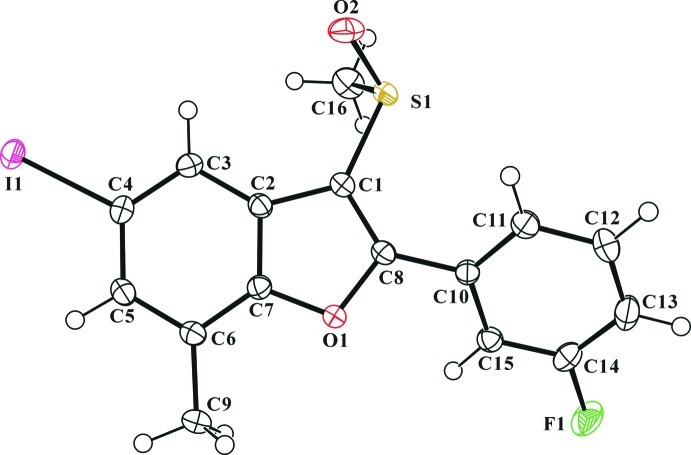
The molecular structure of the title compound with the atom numbering scheme. Displacement ellipsoids are drawn at the 50% probability level. H atoms are presented as small spheres of arbitrary radius.

**Fig. 2.**
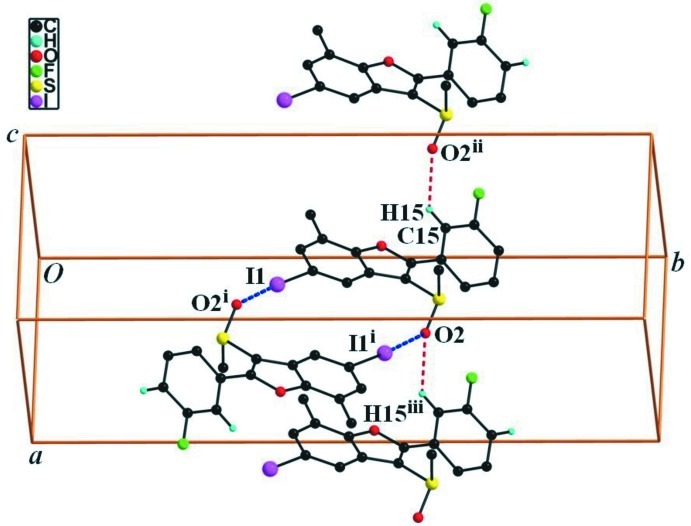
A view of the C—H···O and I···O interactions (dotted lines) in the crystal structure of the title compound. H atoms non-participating in hydrogen-bonding were omitted for clarity. [Symmetry codes: (i) - x + 2, - y + 1, - z + 2; (ii) x - 1, y, z; (iii) x + 1, y, z.]

## supplementary crystallographic information

### Comment

As a part of our ongoing study of 5-iodo-7-methyl-3-methylsulfinyl-1-benzofuran derivatives containing 2-phenyl (Choi *et al.*, 2008) and 2-(4-fluorophenyl) (Choi *et al.*, 2010) substituents, we report herein the crystal structure of the title compound.

In the title molecule (Fig. 1), the benzofuran unit is essentially planar, with a mean deviation of 0.019 (1) Å from the least-squares plane defined by the nine constituent atoms. The dihedral angle between the 3-fluorophenyl ring and the mean plane of the benzofuran fragment is 34.93 (7)°. In the crystal structure (Fig. 2), molecules are linked via pairs of I···O halogen-bondings (Politzer *et al.*, 2007) between the iodine atom and the O atom of the S═O unit [I1···O2^i^ = 3.088 (2) Å, C4—I1···O2^i^ = 169.86 (6)°], forming inversion dimers. These dimers are connected by weak intermolecular C—H···O hydrogen bonds (Table 1).

### Experimental

3-Chloroperoxybenzoic acid (77%, 202 mg, 0.9 mmol) was added in small portions to a stirred solution of 2-(3-fluorophenyl)-5-iodo-7-methyl-3-methylsulfanyl-1-benzofuran (326 mg, 0.8 mmol) in dichloromethane (30 mL) at 273 K. After being stirred at room temperature for 4h, the mixture was washed with saturated sodium bicarbonate solution and the organic layer was separated, dried over magnesium sulfate, filtered and concentrated at reduced pressure. The residue was purified by column chromatography (hexane/ethyl acetate, 1:1 v/v) to afford the title compound as a colorless solid [yield 70%, m.p. 459–460 K; *R*_f_ = 0.56 (hexane?ethyl acetate, 1:1 v/v)]. Single crystals suitable for X-ray diffraction were prepared by slow evaporation of a solution of the title compound in ethyl acetate at room temperature.

### Refinement

All H atoms were positioned geometrically and refined using a riding model, with C—H = 0.95 Å for aryl and 0.98 Å for methyl H atoms. U_iso_(H) was set to 1.2*U*_eq_(C) for aryl and 1.5*U*_eq_(C) for methyl H atoms. The positions of methyl hydrogens were optimized rotationally.

### Crystal data

**Table:**

C_16_H_12_FIO_2_S	*F*(000) = 808
*M**_r_* = 414.22	*D*_x_ = 1.849 Mg m^−^^3^
Monoclinic, *P*2_1_/*c*	Mo *K*α radiation, λ = 0.71073 Å
Hall symbol: -P 2ybc	Cell parameters from 8987 reflections
*a* = 7.8169 (1) Å	θ = 2.7–27.5°
*b* = 23.9721 (3) Å	µ = 2.30 mm^−^^1^
*c* = 8.0559 (1) Å	*T* = 173 K
β = 99.748 (1)°	Block, colourless
*V* = 1487.78 (3) Å^3^	0.30 × 0.22 × 0.19 mm
*Z* = 4	

### Data collection

**Table:**

Bruker SMART APEXII CCD diffractometer	3424 independent reflections
Radiation source: rotating anode	3226 reflections with *I* > 2σ(*I*)
Graphite multilayer monochromator	*R*_int_ = 0.025
Detector resolution: 10.0 pixels mm^-1^	θ_max_ = 27.5°, θ_min_ = 1.7°
φ and ω scans	*h* = −10→8
Absorption correction: multi-scan (*SADABS*; Bruker, 2009)	*k* = −31→30
*T*_min_ = 0.570, *T*_max_ = 0.746	*l* = −10→10
14066 measured reflections	

### Refinement

**Table:**

Refinement on *F*^2^	Primary atom site location: structure-invariant direct methods
Least-squares matrix: full	Secondary atom site location: difference Fourier map
*R*[*F*^2^ > 2σ(*F*^2^)] = 0.021	Hydrogen site location: difference Fourier map
*wR*(*F*^2^) = 0.052	H-atom parameters constrained
*S* = 1.10	*w* = 1/[σ^2^(*F*_o_^2^) + (0.0225*P*)^2^ + 1.0726*P*] where *P* = (*F*_o_^2^ + 2*F*_c_^2^)/3
3424 reflections	(Δ/σ)_max_ = 0.002
192 parameters	Δρ_max_ = 0.45 e Å^−^^3^
0 restraints	Δρ_min_ = −0.55 e Å^−^^3^

### Special details

**Table:**

Geometry. All esds (except the esd in the dihedral angle between two l.s. planes) are estimated using the full covariance matrix. The cell esds are taken into account individually in the estimation of esds in distances, angles and torsion angles; correlations between esds in cell parameters are only used when they are defined by crystal symmetry. An approximate (isotropic) treatment of cell esds is used for estimating esds involving l.s. planes.
Refinement. Refinement of F^2^ against ALL reflections. The weighted R-factor wR and goodness of fit S are based on F^2^, conventional R-factors R are based on F, with F set to zero for negative F^2^. The threshold expression of F^2^ > 2sigma(F^2^) is used only for calculating R-factors(gt) etc. and is not relevant to the choice of reflections for refinement. R-factors based on F^2^ are statistically about twice as large as those based on F, and R- factors based on ALL data will be even larger.

### Fractional atomic coordinates and isotropic or equivalent isotropic displacement parameters (Å^2^)

**Table:**

	*x*	*y*	*z*	*U*_iso_*/*U*_eq_	
I1	0.830625 (17)	0.412987 (6)	1.026115 (17)	0.02457 (6)	
S1	0.70761 (6)	0.66239 (2)	0.72019 (6)	0.02102 (11)	
F1	−0.11935 (17)	0.70760 (6)	0.33734 (19)	0.0382 (3)	
O1	0.31305 (17)	0.56234 (6)	0.56108 (17)	0.0187 (3)	
O2	0.88641 (19)	0.64247 (7)	0.7119 (2)	0.0298 (3)	
C1	0.5622 (3)	0.60638 (8)	0.6682 (2)	0.0192 (4)	
C2	0.5730 (2)	0.54990 (8)	0.7341 (2)	0.0185 (4)	
C3	0.6967 (3)	0.51834 (8)	0.8398 (3)	0.0212 (4)	
H3	0.8058	0.5336	0.8886	0.025*	
C4	0.6524 (3)	0.46397 (8)	0.8699 (2)	0.0204 (4)	
C5	0.4901 (3)	0.44112 (8)	0.8021 (2)	0.0207 (4)	
H5	0.4652	0.4037	0.8283	0.025*	
C6	0.3652 (2)	0.47188 (8)	0.6978 (2)	0.0193 (4)	
C7	0.4151 (2)	0.52550 (8)	0.6654 (2)	0.0182 (4)	
C8	0.4067 (2)	0.61080 (8)	0.5635 (2)	0.0177 (4)	
C9	0.1873 (3)	0.44943 (9)	0.6296 (3)	0.0272 (5)	
H9A	0.1000	0.4706	0.6774	0.041*	
H9B	0.1813	0.4100	0.6603	0.041*	
H9C	0.1648	0.4531	0.5067	0.041*	
C10	0.3182 (2)	0.65543 (8)	0.4579 (2)	0.0184 (4)	
C11	0.4081 (3)	0.69255 (9)	0.3707 (3)	0.0240 (4)	
H11	0.5301	0.6890	0.3772	0.029*	
C12	0.3186 (3)	0.73475 (10)	0.2744 (3)	0.0301 (5)	
H12	0.3802	0.7600	0.2153	0.036*	
C13	0.1406 (3)	0.74046 (10)	0.2636 (3)	0.0300 (5)	
H13	0.0792	0.7698	0.2001	0.036*	
C14	0.0562 (3)	0.70246 (9)	0.3474 (3)	0.0253 (4)	
C15	0.1381 (3)	0.65996 (9)	0.4442 (2)	0.0203 (4)	
H15	0.0743	0.6344	0.5002	0.024*	
C16	0.6965 (3)	0.66712 (10)	0.9404 (3)	0.0308 (5)	
H16A	0.7382	0.6322	0.9964	0.046*	
H16B	0.5760	0.6736	0.9545	0.046*	
H16C	0.7691	0.6981	0.9905	0.046*	

### Atomic displacement parameters (Å^2^)

**Table:**

	*U*^11^	*U*^22^	*U*^33^	*U*^12^	*U*^13^	*U*^23^
I1	0.02277 (8)	0.02225 (8)	0.02801 (8)	0.00522 (5)	0.00230 (6)	0.00455 (5)
S1	0.0205 (2)	0.0182 (2)	0.0234 (2)	−0.00496 (18)	0.00100 (19)	0.00187 (18)
F1	0.0224 (7)	0.0458 (9)	0.0453 (8)	0.0121 (6)	0.0022 (6)	0.0103 (7)
O1	0.0175 (6)	0.0161 (7)	0.0213 (6)	−0.0019 (5)	0.0002 (5)	0.0015 (5)
O2	0.0183 (7)	0.0381 (9)	0.0330 (8)	−0.0056 (6)	0.0044 (6)	−0.0003 (7)
C1	0.0189 (9)	0.0177 (9)	0.0211 (9)	−0.0020 (7)	0.0033 (7)	0.0010 (7)
C2	0.0185 (9)	0.0158 (9)	0.0215 (9)	−0.0006 (7)	0.0045 (7)	−0.0007 (7)
C3	0.0166 (9)	0.0213 (10)	0.0250 (10)	−0.0002 (7)	0.0018 (8)	−0.0002 (8)
C4	0.0210 (9)	0.0201 (10)	0.0201 (9)	0.0040 (7)	0.0033 (8)	0.0024 (7)
C5	0.0224 (10)	0.0163 (10)	0.0246 (10)	−0.0008 (7)	0.0071 (8)	0.0002 (7)
C6	0.0191 (9)	0.0180 (10)	0.0211 (9)	−0.0021 (7)	0.0040 (7)	−0.0020 (7)
C7	0.0169 (9)	0.0185 (10)	0.0191 (9)	0.0004 (7)	0.0032 (7)	0.0007 (7)
C8	0.0177 (9)	0.0168 (9)	0.0193 (9)	−0.0019 (7)	0.0050 (7)	0.0000 (7)
C9	0.0212 (10)	0.0232 (11)	0.0353 (11)	−0.0064 (8)	−0.0002 (9)	0.0009 (9)
C10	0.0203 (9)	0.0169 (9)	0.0173 (9)	−0.0003 (7)	0.0012 (7)	−0.0003 (7)
C11	0.0222 (10)	0.0252 (11)	0.0249 (10)	−0.0013 (8)	0.0050 (8)	0.0026 (8)
C12	0.0346 (12)	0.0257 (11)	0.0305 (11)	−0.0036 (9)	0.0070 (9)	0.0091 (9)
C13	0.0361 (12)	0.0248 (11)	0.0274 (10)	0.0060 (9)	0.0004 (9)	0.0068 (9)
C14	0.0213 (10)	0.0283 (11)	0.0251 (10)	0.0064 (8)	0.0010 (8)	−0.0002 (8)
C15	0.0210 (9)	0.0198 (10)	0.0200 (9)	−0.0009 (7)	0.0030 (7)	−0.0011 (7)
C16	0.0372 (12)	0.0309 (12)	0.0245 (10)	−0.0044 (10)	0.0063 (9)	−0.0049 (9)

### Geometric parameters (Å, º)

**Table:**

I1—C4	2.1034 (19)	C6—C9	1.505 (3)
I1—O2^i^	3.0876 (16)	C8—C10	1.466 (3)
S1—O2	1.4891 (16)	C9—H9A	0.9800
S1—C1	1.763 (2)	C9—H9B	0.9800
S1—C16	1.795 (2)	C9—H9C	0.9800
F1—C14	1.366 (2)	C10—C11	1.395 (3)
O1—C8	1.371 (2)	C10—C15	1.398 (3)
O1—C7	1.376 (2)	C11—C12	1.390 (3)
C1—C8	1.361 (3)	C11—H11	0.9500
C1—C2	1.451 (3)	C12—C13	1.386 (3)
C2—C7	1.393 (3)	C12—H12	0.9500
C2—C3	1.397 (3)	C13—C14	1.368 (3)
C3—C4	1.380 (3)	C13—H13	0.9500
C3—H3	0.9500	C14—C15	1.374 (3)
C4—C5	1.405 (3)	C15—H15	0.9500
C5—C6	1.388 (3)	C16—H16A	0.9800
C5—H5	0.9500	C16—H16B	0.9800
C6—C7	1.381 (3)	C16—H16C	0.9800
			
C4—I1—O2^i^	169.86 (6)	C6—C9—H9B	109.5
O2—S1—C1	108.51 (9)	H9A—C9—H9B	109.5
O2—S1—C16	105.52 (10)	C6—C9—H9C	109.5
C1—S1—C16	98.38 (10)	H9A—C9—H9C	109.5
C8—O1—C7	106.39 (14)	H9B—C9—H9C	109.5
C8—C1—C2	106.86 (17)	C11—C10—C15	119.85 (18)
C8—C1—S1	124.05 (15)	C11—C10—C8	121.95 (18)
C2—C1—S1	128.91 (14)	C15—C10—C8	118.20 (17)
C7—C2—C3	119.33 (18)	C12—C11—C10	119.7 (2)
C7—C2—C1	104.67 (16)	C12—C11—H11	120.1
C3—C2—C1	136.00 (18)	C10—C11—H11	120.1
C4—C3—C2	116.76 (18)	C13—C12—C11	120.8 (2)
C4—C3—H3	121.6	C13—C12—H12	119.6
C2—C3—H3	121.6	C11—C12—H12	119.6
C3—C4—C5	122.44 (18)	C14—C13—C12	117.8 (2)
C3—C4—I1	119.57 (14)	C14—C13—H13	121.1
C5—C4—I1	117.98 (15)	C12—C13—H13	121.1
C6—C5—C4	121.68 (19)	F1—C14—C13	118.30 (19)
C6—C5—H5	119.2	F1—C14—C15	117.87 (19)
C4—C5—H5	119.2	C13—C14—C15	123.8 (2)
C7—C6—C5	114.62 (18)	C14—C15—C10	117.96 (19)
C7—C6—C9	122.47 (18)	C14—C15—H15	121.0
C5—C6—C9	122.89 (19)	C10—C15—H15	121.0
O1—C7—C6	124.02 (17)	S1—C16—H16A	109.5
O1—C7—C2	110.87 (17)	S1—C16—H16B	109.5
C6—C7—C2	125.11 (18)	H16A—C16—H16B	109.5
C1—C8—O1	111.17 (17)	S1—C16—H16C	109.5
C1—C8—C10	134.49 (18)	H16A—C16—H16C	109.5
O1—C8—C10	114.33 (16)	H16B—C16—H16C	109.5
C6—C9—H9A	109.5		
			
O2—S1—C1—C8	−136.08 (17)	C3—C2—C7—O1	−178.14 (17)
C16—S1—C1—C8	114.37 (19)	C1—C2—C7—O1	1.4 (2)
O2—S1—C1—C2	49.5 (2)	C3—C2—C7—C6	2.5 (3)
C16—S1—C1—C2	−60.0 (2)	C1—C2—C7—C6	−177.92 (19)
C8—C1—C2—C7	−2.2 (2)	C2—C1—C8—O1	2.2 (2)
S1—C1—C2—C7	172.98 (16)	S1—C1—C8—O1	−173.22 (14)
C8—C1—C2—C3	177.3 (2)	C2—C1—C8—C10	−179.2 (2)
S1—C1—C2—C3	−7.6 (4)	S1—C1—C8—C10	5.3 (3)
C7—C2—C3—C4	−0.2 (3)	C7—O1—C8—C1	−1.4 (2)
C1—C2—C3—C4	−179.6 (2)	C7—O1—C8—C10	179.76 (16)
C2—C3—C4—C5	−1.5 (3)	C1—C8—C10—C11	37.1 (3)
C2—C3—C4—I1	179.60 (14)	O1—C8—C10—C11	−144.37 (18)
O2^i^—I1—C4—C3	31.2 (5)	C1—C8—C10—C15	−143.5 (2)
O2^i^—I1—C4—C5	−147.7 (3)	O1—C8—C10—C15	35.0 (2)
C3—C4—C5—C6	1.1 (3)	C15—C10—C11—C12	1.7 (3)
I1—C4—C5—C6	−179.97 (15)	C8—C10—C11—C12	−178.94 (19)
C4—C5—C6—C7	1.0 (3)	C10—C11—C12—C13	−0.1 (3)
C4—C5—C6—C9	−177.2 (2)	C11—C12—C13—C14	−1.5 (3)
C8—O1—C7—C6	179.23 (18)	C12—C13—C14—F1	−179.9 (2)
C8—O1—C7—C2	−0.1 (2)	C12—C13—C14—C15	1.5 (3)
C5—C6—C7—O1	177.90 (18)	F1—C14—C15—C10	−178.52 (18)
C9—C6—C7—O1	−3.9 (3)	C13—C14—C15—C10	0.1 (3)
C5—C6—C7—C2	−2.8 (3)	C11—C10—C15—C14	−1.7 (3)
C9—C6—C7—C2	175.3 (2)	C8—C10—C15—C14	178.90 (18)

Symmetry code: (i) −*x*+2, −*y*+1, −*z*+2.

### Hydrogen-bond geometry (Å, º)

**Table:**

*D*—H···*A*	*D*—H	H···*A*	*D*···*A*	*D*—H···*A*
C15—H15···O2^ii^	0.95	2.44	3.183 (3)	135

Symmetry code: (ii) *x*−1, *y*, *z*.
